# Near-Infrared Spectroscopy and Aquaphotomics for Monitoring Mung Bean (*Vigna radiata*) Sprout Growth and Validation of Ascorbic Acid Content

**DOI:** 10.3390/s21020611

**Published:** 2021-01-17

**Authors:** David Tjandra Nugraha, John-Lewis Zinia Zaukuu, Juan Pablo Aguinaga Bósquez, Zsanett Bodor, Flora Vitalis, Zoltan Kovacs

**Affiliations:** Institute of Bioengineering and Process Control, Department of Measurements and Process Control, Faculty of Food Science, Szent István University, 1118 Budapest, Hungary; tjandra.nugraha.david@hallgato.uni-szie.hu (D.T.N.); zaukuu.john-lewis.zinia@hallgato.uni-szie.hu (J.-L.Z.Z.); aguinaga.bosquez.juan.pablo@hallgato.uni-szie.hu (J.P.A.B.); bodor.zsanett@hallgato.uni-szie.hu (Z.B.); vitalis.flora@hallgato.uni-szie.hu (F.V.)

**Keywords:** germination, water spectral pattern, chemometrics, malting

## Abstract

Mung bean is a leguminous crop with specific trait in its diet, namely in the form of anti-nutrient components. The sprouting process is commonly done for better nutritional acceptance of mung bean as it presents better nutritional benefits. Sprouted mung bean serves as a cheap source of protein and ascorbic acid, which are dependent on the sprouting process, hence the importance of following the biological process. In larger production scale, there has not been a definite standard for mung bean sprouting, raising the need for quick and effective mung bean sprout quality checks. In this regard, near-infrared spectroscopy (NIRS) has been recognized as a highly sensitive technique for quality control that seems suitable for this study. The aim of this paper was to describe quality parameters (water content, pH, conductivity, and ascorbic acid by titration) during sprouting using conventional analytical methods and advanced NIRS techniques as correlative methods for modelling sprouted mung beans’ quality and ascorbic acid content. Mung beans were sprouted in 6 h intervals up to 120 h and analyzed using conventional methods and a NIR instrument. The results of the standard analytical methods were analyzed with univariate statistics (analysis of variance (ANOVA)), and the NIRS spectral data was assessed with the chemometrics approach (principal component analysis (PCA), discriminant analysis (DA), and partial least squares regression (PLSR)). Water content showed a monotonous increase during the 120 h of sprouting. The change in pH and conductivity did not describe a clear pattern during the sprouting, confirming the complexity of the biological process. Spectral data-based discriminant analysis was able to distinctly classify the bean sprouts with 100% prediction accuracy. A NIRS-based model for ascorbic acid determination was made using standard ascorbic acid to quantify the components in the bean extract. A rapid detection technique within sub-percent level was developed for mung bean ascorbic acid content with R^2^ above 0.90. The NIR-based prediction offers reliable estimation of mung bean sprout quality

## 1. Introduction

Mung bean (*Vigna radiata*) is one of the most important commodities in Asia. It plays an important role as a cheap source of protein in cereal-based diets. The seeds are usually eaten whole, cooked or fermented, or milled into a flour [1,2]. The application of the flour is diverse and can be used to make noodles, bread, and other bakery products [3,4,5,6]. Mung bean generally contains significant amounts of protein, ranging from 18% to 36%. Apart from having considerable amounts of protein, the bean contains useful nutrients such as fiber, soluble fiber, potassium, and vitamins [7]. The beans are regarded to have low amounts of fat, cholesterol, and sodium. The phosphorous content is significant but the molecules come in the form of phytate, an anti-nutrient component [8,9]. This component has shown a binding activity with divalent cations such as zinc, calcium, magnesium, and iron, creating insoluble compounds [10]. While this is one of the causes for the low mineral bioavailability in the bean, some processes such as germination, soaking, fermentation, and cooking have all proven to reduce the effect of phytate components in mung bean [11,12].

In China and the United States, mung beans are commonly found in their sprouted form. Dried seeds of the bean have relatively long shelf life, and germination can be done at any point. Normally, the sprouting process can be done in a dark environment for up to four days [7], leading to high water content in the bean sprout that makes it pleasurable to eat. As such, they are often used as a garnish in many diets. Besides consumption purposes, sprouting is considered beneficial from the perspective of nutritional value [13,14]. Sprouting has been acknowledged as a process to diminish anti-nutrient components such as phytate and trypsin inhibitor. The biological process also increases the enzyme activity, reducing the amount of flatulence-related oligosaccharides [15]. Complex biochemical processes produce a lot of functional components that increase the overall nutritional value of sprouted bean compared with other types of processing. Notable components are phenolic components and vitamins, especially ascorbic acid. Many researchers have reported different values for ascorbic acid in sprouted mung bean, but it is deemed as one of the most relevant components from the germination process [16,17,18]. Although there are numerous setups for mung bean sprouting, there has not been any specific quality parameter defined for the characterization of the sprouting. In this regard, a rapid and reliable observation method can be developed to monitor the optimal sprouting time based on the best nutritional composition of the bean. Generally, the beans for consumption are served after being germinated for 72–120 h [7,16]. Among the many nutritional parameters of the sprouted beans, ascorbic acid can be considered as one of the most important. Aside from the other nutritional benefits from the sprouting, ascorbic acid formation is significantly affected by the germination time. Its initial content has been reported to be as low as 3 mg/100 g, and the final content of ascorbic acid post-germination can go as high as 98 mg/100 g [19,20,21,22,23]. The significant increase of this component can be a good reason to monitor the germination time from a nutritional perspective.

An objective evaluation method for the rapid determination of the optimal mung bean germination time is highly required. Several selected quality components will be assessed to differentiate the germination time, while special attention will be paid to ascorbic acid formation over time. Generally, it is common to measure water content in such products, as significant water-related changes can affect the germination process as a whole [24]. Organic acid formation is also important, as anabolism reactions go hand in hand with the sprouting phenomena [25,26]. Thus, measurement of pH and conductivity can be an alternative to follow the sprouting process. However, these methods are time and reagent consuming. For objective observation, developing rapid analysis that is cheap, efficient, and applicable on an industrial scale is of paramount importance

Near-infrared spectroscopy (NIRS) is a quick, non-destructive analytical method that has been reported to be able to discriminate bean sprouts based on their germination time for quality and functional purposes by measuring the absorbance within the wavelength of 700–2500 nm [27]. Based on the spectral changes, it can be possible to detect differences within samples or groups of samples as the structural and chemical differences reflect back in the NIR spectra [28]. Therefore, by applying multivariate statistics, it is possible to compare different samples with each other [29]. NIRS is a correlative method, which means with this method, we may not be able to observe the exact composition of the sample, but in the possession of the physicochemical data obtained by reference conventional methods, it could be possible to build correlation models to predict the quantity of the constituents and value of physicochemical data such as pH, electrical conductivity, and vitamin content. Moreover, if there is a reference database containing the physicochemical data of the analyte sample group (in our case mung beans) and the NIR spectra, it would be possible to analyze the composition of a sample without measuring the physicochemical data. This is very beneficial because the determination of the constituents or physicochemical parameters is mostly done by techniques that rely on different reactions and in most of the cases, needs reagents. Moreover, these methods are time-consuming and in some cases, can only be done by experts. Therefore, the application of NIR could be a good solution for analyses with less time, and reagents that could also fulfill the requirement of sustainability, which is a highly researched and actual topic nowadays. In combination with multivariate statistics, NIR spectroscopy has also been used to monitor germination in other variants of plants (forage grass) [30]. Other researchers also reported successful attempts to monitor small concentrations of important components from the bean sprouts using NIR spectroscopy [31]. The use of NIR spectroscopy to determine quality parameters such as ascorbic acid concentration has been reported in different commodities with accuracies as low as 20 mg/100 g [32], although it has not been studied in greater depth about the possibility of rapid quantification of ascorbic acid at the sub-percent level. Using NIR spectroscopy, it can be difficult to determine small concentrations of proximate components in living organisms with high variability, however, the possibility to analyze the general sample of the sprouted beans as extracts provides grounds for further research. Utilizing the aquaphotomics method, it is possible to characterize complex aqueous systems through the changes in the hydrogen bonding network of water molecules as a molecular mirror [33]. The approach mainly takes advantage of highly sensitive water hydrogen bonds under the first overtone range of O-H bond (1300–1600 nm) to disclose hidden patterns within the spectral information [34]. This method also makes it possible to eliminate the weakness of NIR spectroscopy related to water that perturbs quantification of low-concentration components [35,36]. Application of chemometrics methods are commonly used in analyzing spectral data to give better interpretation [37]. Shortly said, conventional analytical methods have some disadvantages, where NIR spectroscopy and aquaphotomics can fill in the gap.

The goal of this study was to investigate the possibility of rapid quality evaluation of mung bean sprout through NIR spectroscopy and aquaphotomics. Our objective was to observe the sprouting process, classify the mung bean sprouts based on germination time, and test the robustness of NIR spectroscopy and aquaphotomics in making a correlative model for rapid quality evaluation and ascorbic acid formation within low percentage ranges in germinated mung beans.

## 2. Materials and Methods

### 2.1. Materials

Mung beans (*Vigna radiata*) were purchased from Thailand (Oriental Food Ltd., Bangkok, Thailand) in packages of 400 g. Beans from six packages were thoroughly homogenized before separation into 21 different holders (containing about 100 g of beans each) based on their germination time (0–120 h, with 6 h intervals). The beans were used as a whole, including the root and stem for the grown sprouts. Distilled water was used to dilute the bean extract after crushing for proximate analysis.

### 2.2. Methods

#### 2.2.1. Bean Growing and Sprout Extract Sample Preparation Method

The germination time (sprouting) for the beans was 120 h long, with sampling every 6 h, resulting in 21 germination time points, including 0 h. The beans in each container (100 g) were soaked with 200 mL of tap water. The soaking process was done for 12 h for every germination time point, before draining the water and rinsing with 200 mL of tap water. The beans were grown on two-fold cellulose paper with 30 × 30 cm in size. For every flat plate, the beans were evenly spread and kept in an incubator with constant temperature of 35 ± 1 °C and relative humidity of 65–80%. The beans were rinsed with tap water in 12 h intervals, and the paper was changed.

After reaching the desired germination time (0–120 h in 6 h intervals), the beans were dried using a two-fold dried tissue paper, scanned with a NIR instrument for the intact bean, and processed into a solution, which was used for subsequent measurements (pH, conductivity, and ascorbic acid by titration). For this, 100 g of the dried whole bean sprout was weighed, and mixed with 200 mL of distilled water before crushing with a small crusher, and filtered using two-fold 100% cellulose paper (100 µm pore size). The filtrate was referred to as the bean sprout extract sample and divided into aliquots for different physicochemical (pH, conductivity, ascorbic acid determination) and NIR spectroscopy measurements.

#### 2.2.2. Determination of Water Content

The method for water content determination was based on the AOAC (Association of Official Analytical Chemists) method [38]. Empty dish and lid were dried in an oven at 105 °C for 3 h, and the constant weight of the containers were measured. Using an analytical scale, around 3 g of bean sprout sample was weighed, and put inside the container. The container was then put inside the oven for another 3 h. The water content was then calculated from the mass that was lost during the oven drying. The water content determination was done in triplicate.

#### 2.2.3. Determination of pH and Conductivity

The pH and electrical conductivity of the bean sprout extract samples were determined using a benchtop pH and electrical conductivity meter instrument (SevenMulti, Mettler Toledo, Greifensee, Switzerland). About 30 mL of the bean sprout extract sample was put inside a glass beaker before the measurements. The electrodes were dipped inside the bean sprout extract and were rinsed with distilled water before every reading. The sequence of the measurement was randomized, and every sample was analyzed in triplicate.

#### 2.2.4. Determination of Ascorbic Acid

The ascorbic acid content of bean sprout extract was determined by the modified direct redox titration using iodine solution as the titrant [39]. For the experiment, 0.01 N iodine solution was prepared by diluting iodine crystal (99%) with distilled water. An additional indicator of 10% potassium iodide solution was made by diluting potassium iodide crystal (99%) with distilled water. Sulfuric acid (0.3 M) was used to promote the reaction. The acid was made by diluting 62% sulfuric acid with distilled water. Starch indicator (1% *w*/*v*) was made by diluting starch powder in hot distilled water. The starch solution was mixed and stirred well before usage.

Before the titration, validation of iodine solution is essential, especially when dealing with a low concentration of substance. The titrant (iodine solution) was standardized by titrating it with sodium thiosulfate. As sodium thiosulfate itself is not stable, it was also standardized with a stable potassium iodate solution. For the standardization, 0.01 N potassium iodate was made by diluting potassium iodate crystal (99.5%) with distilled water. Sodium thiosulfate (0.01 N) was made by diluting sodium thiosulfate crystal (98% anhydrous) with CO_2_-free distilled water. The standardization began by titrating 10 mL of sodium thiosulfate added with 2 mL of potassium iodide 10% solution and 1 mL of sulfuric acid (0.3 M) against potassium iodate as the titrant. The titration was continued until the color of the solution turned light yellow, and 1 mL of starch 1% was added. The titration was continued until the solution turned colorless. The volume of the used potassium iodate was recorded, and the titration was done in triplicate. The known exact concentration of the sodium thiosulfate was able to be used as the standard for iodine solution validation and determined as shown in Equation (1). For iodine solution concentration determination, the iodine solution was measured at approximately 10 mL, and titrated with the previously determined sodium thiosulfate. The normality of iodine solution was then determined as shown in Equation (2). In Equations (1) and (2), N means normality and V means volume:

N Sodium Thiosulfate = V Potassium Iodate × N Potassium Iodate × 0.1(1)

N Iodine = V Sodium Thiosulfate × N Sodium Thiosulfate × 0.1(2)

The bean sprout extract was used for the determination of ascorbic acid. Approximately 25 mL of the aliquot was used, and titrated in the same way as the standardization method. The titration was done in triplicate for each bean sprout germination time. The blank solution was 25 mL of distilled water. The volume that was recorded was the subtraction of the sample titration volume and the blank titration volume. The mass of ascorbic acid in the 25 mL aliquot was determined by multiplying the normality of the titrant (iodine solution), titrant volume, and equivalent weight of ascorbic acid against iodine (176.13 divided by 2), as shown in Equation (3), where Mg means mass of ascorbic acid and V means volume:

Mg Ascorbic Acid = N Sodium Thiosulfate × (V sample − V blank) × 88.065(3)

#### 2.2.5. Calibration of NIR Spectroscopy-Based Ascorbic Acid Model

For the calibration of ascorbic acid modelling by NIR spectroscopy, 99% L-ascorbic acid was diluted with known concentration. The range of the ascorbic acid standard curve was based on the concentration of that was suspected to be in the beans based on previous findings [7,16]. The standard calibration curve consisted of six points and the maximum concentration was 500 mg/L or 50 mg/100 mL in 100 mg/L step. This model was used to estimate the ascorbic acid content of the bean sprout extract.

#### 2.2.6. Near-Infrared (NIR) Spectroscopy Measurement of Sprouted Bean and Bean Extracts

NIR spectroscopy measurements were done using both the intact bean sprout samples and the bean sprout extracts. For the intact bean sprout measurement, beans were subjected to NIR spectroscopy measurement every 24 h of germination time point (0–120 h) to test the classification accuracy of classes on a daily basis. Each individual bean sprout was scanned one by one for each germination time with a DLP (Digital Light Processing) NIRScanNano instrument (Texas Instruments, Dallas, TX, USA). Exactly 20 bean samples from each germination time point were scanned in triplicate. The scan was done on the bean part of the sprout, so the grown part was removed for the scan. The spectral data was collected in the wavelength interval of 900–1700 nm by diffuse reflectance mode using a circular cuvette of 4.1” in diameter.

The bean sprout extract samples were also analyzed with the DLP NIRScanNano instrument (Texas Instruments, Dallas, TX, USA) but using a quartz cuvette of 1 mm path length in transmission mode. The scanning was done in triplicate recording five consecutive scans for each sample, resulting in 15 scans for every germination time point. The sequence of the scans was random for all of the NIR spectroscopy measurements. The ascorbic acid standard solutions were scanned using the same instrument, i.e., using a quartz cuvette of 1 mm path length and transmission mode. The scans for each concentration were done in triplicate, with three consecutive scans each.

#### 2.2.7. Statistical Methods

The results of water content, pH, conductivity, and ascorbic acid content determination were analyzed using descriptive statistics (mean, standard deviation (SD)) for initial evaluation. One-way analysis of variance (ANOVA) was used, followed by Duncan’s Multiple Range Test (DMRT) at the *p* < 0.05 confidence interval. One-way ANOVA was used as a method to evaluate the significance of the germination time for the dependent variables. The same superscript indicates no significant difference within the sprouting times, while different letters indicate a significant difference [40]. Durbin-Watson statistic was used to detect autocorrelation in the models before plotting the water content and ascorbic acid result.

Multivariate statistics were used to interpret the NIR spectroscopy spectral data and to build classification and regression models. After raw spectra analysis, the spectral range of the data from the intact bean sprout and bean sprout extract measurement were truncated in a 950–1630 nm interval to cut off noisy ends of the spectrum. Those from the bean sprout extract were further truncated in the range 1300–1600 nm for the aquaphotomics analysis. The spectral data from both datasets were pre-treated to reduce the spectral noise and undesired baseline variations occurred by scattering. Savitzky-Golay (SG) smoothing with second-order polynomial and 15 points was used to reduce noise of the data without disturbing the signal tendencies [41], and standard normal variate (SNV) was applied to correct multiplicative interferences of light scatter [42].

The analysis of intact bean and bean sprout extract spectra were done separately. Representation of the classes based on principal component analysis (PCA) was done to show outliers within the dataset, as well as identifying the underlying pattern. The linear discriminant analysis (LDA) models were built based on the first ten principal component (PC) scores to maximize between-class separation, and minimize within-class separation. LDA models were built separately for the intact bean and bean sprout extract based on each germination time point. Predictive capability of the LDA model was tested by dividing the dataset into calibration data (training) and validation data (prediction). The calibration data firstly contained two-thirds of the data, consisting of the first and second replicate. The third replicate was tested against this calibration data. The validation was done in three-fold, to ensure that each replicate was used for calibration and validation at least once. Finally, the average accuracy of recognition and prediction for the cross-validation were used to characterize the accuracy of the classification models. The important wavelengths were determined from PCA, where wavelengths contributing for PC1 and PC2 were selected for quantitative modelling. Partial least squares regression (PLSR) was used to predict water content, germination time, and ascorbic acid content based on the NIR spectra of the bean extracts. The spectral data of the intact bean was not used, because the methods to determine quality parameters for single kernel are not applicable, thus lacking in the reference data needed for predictive models. For all of the predictions, the dataset was divided into calibration and validation once again, and k-fold cross-validation was used to test the predictive accuracy. For the cross-validation, the spectral data of the same germination time point were left out. Coefficient of determination (R^2^) value was given, and root means square error (RMSE) of the calibration (C) and validation (CV) was used to determine the optimal number of latent variables used for the PLSR model to prevent overfitting.

For the aquaphotomics method, an aquagram was calculated from the spectral data. Aquagram is a star-shaped diagram which shows normalized and averaged absorbance at the selected wavelengths according to specific water absorbance bands [43]. It can be an effective tool to examine the dynamics of water structural changes along some perturbation [33], like in this study, where it was used to analyze changes in water structure in the germinated bean during the sprouting period (0–120 h).

## 3. Results

### 3.1. Results of Conventional Methods

#### 3.1.1. Results of Water Content Determination

**Figure 1:**
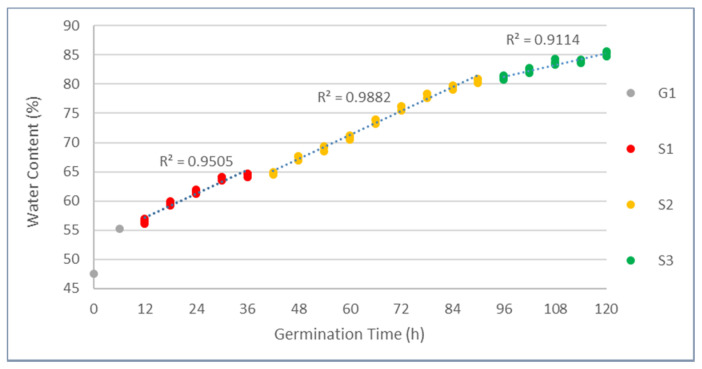
Water content plot of mung bean sprout in the function of germination time.

The water content plot is segmented in distinct colors per different growth stages. The first growth phase was defined when the sprout growth was not significant (G1): the dominant part of the beans remained within the cotyledon, and the sprout could hardly be plucked from the cotyledon. The second growth phase (S1, S2, and S3) was defined where part of the sprout had grown significantly, and the outer membrane of the seed was completely broken. The roots also started to form complex branching structures from the end of the sprout (S3). The change of water content in the function of time (Figure 1) showed a monotonic increase with transitions between the different stages of growth. The results of the ANOVA test showed a significant effect of germination time in the water content of the sprouted beans (*p* < 0.05). The post-hoc test revealed that each of the germination time points were significantly different from each other, denoting discrete annotations for each of the water content results based on DMRT (Table 1).

#### 3.1.2. Results of pH and Conductivity Determination

The values from pH and conductivity are summarized in Table 1. While the change in pH parameter is more straightforward, when measuring the hydrogen ion within the solution, conductivity can be affected by molecular properties such as the morphology of the molecules and the amount of soluble contents within the liquid [44]. The results of the ANOVA (Table 1) showed that germination time showed a significant effect on both the pH and conductivity of the bean sprout extract sample (*p* < 0.05). The post-hoc DMRT test showed that most of the germination time points differ, but the trend from pH and conductivity measurement did not show any similar tendency.

### 3.2. Results of Near-Infrared Spectroscopic Analysis

#### 3.2.1. Results of Mung Bean Sprout Identification

**Figure 2:**
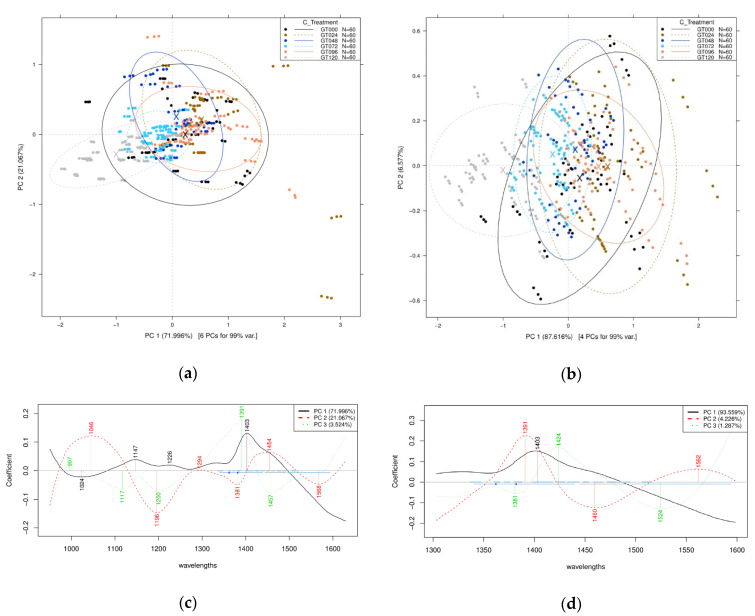
Principal component analysis (PCA) of germination time on the entire Near-Infrared Spectroscopy (NIRS) spectral data of the mung bean sprout after Savitzky-Golay smoothing (SG) and standard normal variate (SNV) pretreatment of the spectra: (**a**) Score plot within 950–1630 nm wavelength interval, (**b**) score plot within 1300–1600 nm first O-H overtone wavelength, (**c**) principal components (PC) loadings plot within 950–1630 nm wavelength interval, and (**d**) PC loadings plot within 1300–1600 nm wavelength interval.

The PCA plot of the intact bean scans can be seen in Figure 2a,b, where it does not show a specific tendency.

The results from the PCA for the wider wavelength interval showed 93.03% of the total variance described by the first two PCs (PC1 and PC2), while the PCA for the first overtone wavelength range showed 94.19% of the same PCs. From the PCA of the 950–1630 nm wavelength, it can be seen that the spectral characteristics of raw mung bean (0 h) were dominant and the grouping could hardly determine the difference between the 0 h and the other germination time points (Figure 2a). In the first overtone wavelength interval (1300–1600 nm), the characteristics can be differentiated, although 0 h once again showed dominant characteristics in the groups (Figure 2b). With the model built on the 950–1630 nm spectral range, the following wavelengths were identified as the main contributors to PC1 and PC2: 1024, 1147, 1226, 1403 nm, and 1046, 1196, 1294, 1381, 1454, and 1568 nm (Figure 2c). In the first overtone region, 1403 and 1391 nm were found to be important in the formation of PC1 and PC2, respectively (Figure 2d).

**Figure 3:**
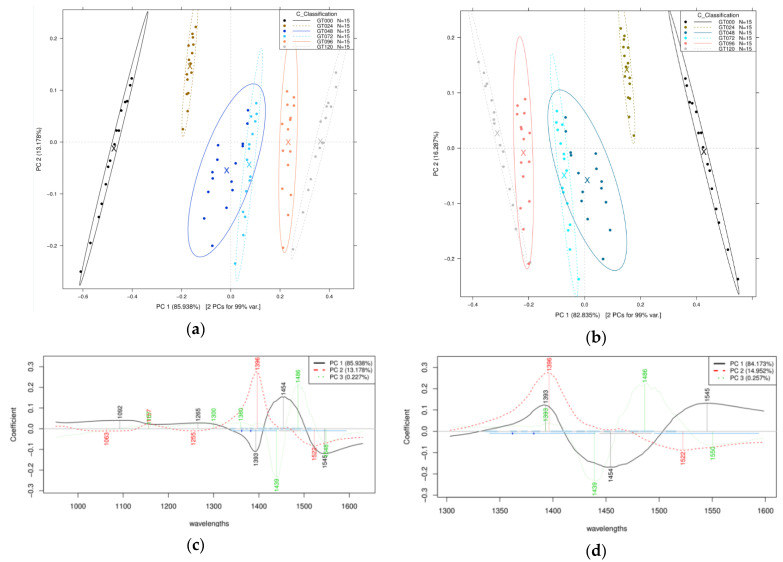
Principal component analysis (PCA) on the entire Near-Infrared Spectroscopy (NIRS) spectral data of the mung bean sprout extract after Savitzky-Golay smoothing (SG) and standard normal variate (SNV) pretreatment of the spectra: (**a**) Score plot within 950–1630 nm wavelength interval, (**b**) score plot within 1300–1600 nm first O-H overtone wavelength, (**c**) principal components (PC) loadings plot within 950–1630 nm wavelength interval, and (**d**) PC loadings plot within 1300–1600 nm wavelength interval.

The PCA plot of the NIR spectroscopy measurement from the bean sprout extract can be seen in Figure 3. The results from the wider wavelength interval PCA described 99.11% of the total variance described by the first two PCs, while the results of the first overtone wavelength described 99.12% of the total variance by the same PCs. In the case of both PCA models, a separation tendency can be seen through PC1 according to the time of germination, while PC2 mostly showed the deviation within the groups of the same germination time (Figure 3a,b). The model built for the whole range of the spectra showed that wavelengths of 1092, 1265, 1393, 1454, and 1545 nm contributed to the formation of PC1. While in the formation of PC2, 1063, 1255, 1369, and 1522 nm had the highest role (Figure 3c). Results of the model built for the range of first overtone showed similar results as the same wavelengths contributed to the PC1 and PC2 (Figure 3d).

**Figure 4:**
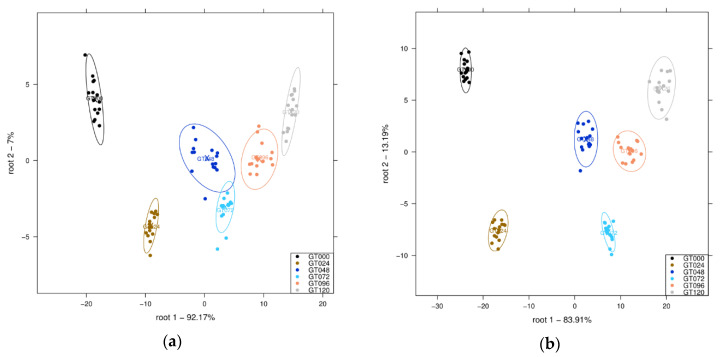
Linear discriminant analysis (LDA) on the spectral data of the mung bean sprout extract after Savitzky-Golay smoothing (SG) and standard normal variate (SNV) pretreatment, and three-fold cross-validation: (**a**) Score plot within 950–1630 nm wavelength interval, and (**b**) score plot within 1300–1600 nm first O-H overtone wavelength.

The LDA model built for sprouting time classification can be seen in Figure 4. Both plots presenting results of the wider spectral range (Figure 4a) and the range of the first overtone (Figure 4b) showed similar tendency of the separation based on root 1. Although more of the classification could be described from the analysis using the wider spectral data (950–1630 nm), the narrow wavelength interval showed better within-class intervals that created grouped clusters. There was 100% correct accuracy after cross-validation, meaning there were no false predictions of sprouting time from mung bean within the 24 h experimented time interval. There was no significant difference within the scope of the analysis, so further analysis was performed on the narrower wavelength interval, complying with the aquaphotomics method.

The important wavelengths from the NIR spectroscopy scans have been summarized and tabulated for easier comparison (Table 2).

**Figure 5:**
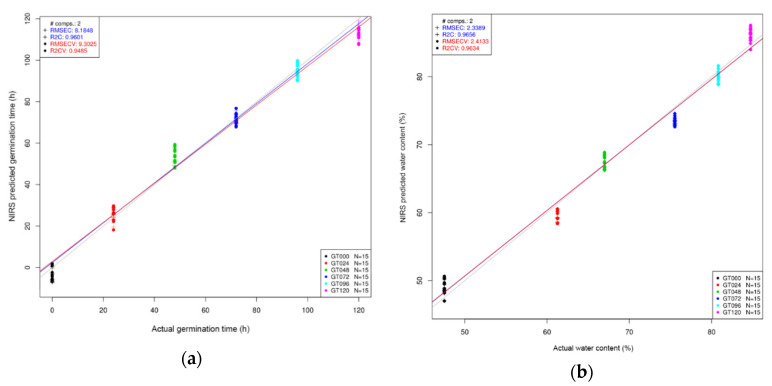
Partial least squares regression (PLSR) of bean sprout extract spectral data in the wavelength interval of 1300–1600 nm, after Savitzky-Golay smoothing (SG) and standard normal variate (SNV) pretreatment of the spectra and 6-fold cross-validation method: (**a**) Regression based on germination time of the sprouts, and (**b**) regression based on the water content of the sprouts.

Predictive models of germination time and water content can be seen in Figure 5, where the coefficient of determination (R^2^) for both models was satisfactory. The prediction model was based only on the important wavelengths of bean sprout extract sample (Table 2). The R^2^ for sprouting time prediction was 0.9601 but was 0.9485 after cross-validation with root mean square error of calibration (RMSEC) of 8.1848 and root mean square error of cross-validation (RMSECV) of 9.3025. For the water content prediction, the R^2^ was 0.9656 and 0.9634 after cross-validation with RMSEC of 2.3389 and RMSECV of 2.4133. The number of samples was the same (N = 90), and it used two variables respectively for both predictions. The regression vector showed that the wavelength 1454 nm was the most important wavelength for both water content and germination prediction (Figure 5c).

#### 3.2.2. Results of Ascorbic Acid Modelling

**Figure 6:**
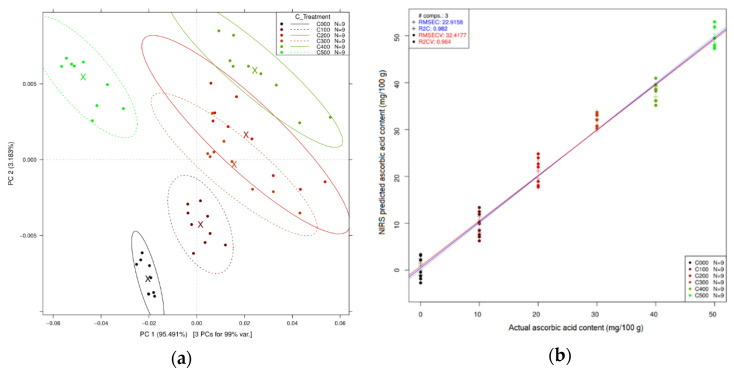

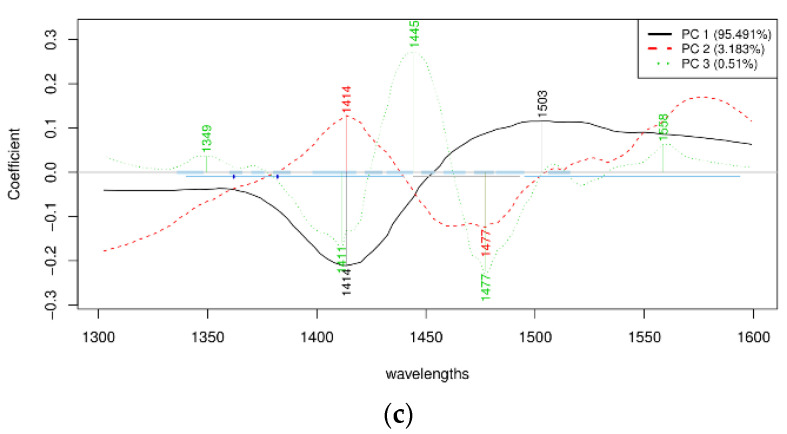
Multivariate analysis from the ascorbic acid spectral data after SG and SNV pretreatment: (**a**) PCA plot of ascorbic acid within 1300–1600 nm wavelength, (**b**) PLSR plot of ascorbic acid after 6-fold cross-validation, and (**c**) PCs loading plot of ascorbic acid.

Multivariate analysis from the ascorbic acid NIR spectroscopy analysis can be seen in Figure 6. From the PCA plot, a monotone separation trend could be observed based on different concentrations through PC1 and PC2, which described 95.491% and 3.183% of the total variance, respectively. Although variability within-class could also be seen, it did not hinder the classification of the classes. For the formation of PC1, the wavelengths of 1414 and 1503 nm contributed the most. For the formation of PC2, it was 1414 and 1477 nm. The PLSR plot itself showed satisfactory results with 0.982 R^2^, and 0.964 after the cross-validation (R^2^CV). The RMSEC is 22.9158, and the RMSECV is 32.4177. The PLSR model regression vector showed that 1387, 1411, 1441, 1471, 1501, and 1547 nm contributed the most to the prediction of ascorbic acid concentration.

**Figure 7:**
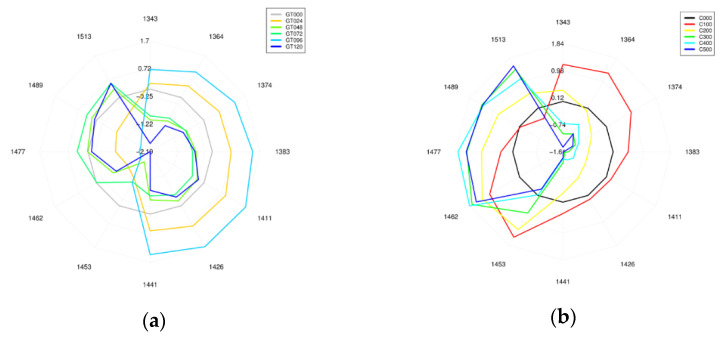
Aquagram plot in the wavelength interval of 1300–1600 nm, after Savitzky-Golay smoothing (SG) and standard normal variate (SNV) pretreatment of the spectra: (**a**) Aquagram plot of germinated mung bean sprout with 24 h intervals, and (**b**) aquagram plot of ascorbic acid with different concentrations.

Aquagram plots of the bean sprout extract and ascorbic acid are presented in Figure 7. As the aim of this study was to predict one of the essential components of germination (e.g., ascorbic acid), comparison between these two is important. Both aquagrams showed similarity in trend. Both mung bean sprout and ascorbic acid initially showed weaker bonded water molecule (first and fourth quadrant), and stronger bonded water (1513 nm) was observed under higher concentration of ascorbic acid, and longer germination time.

From Table 3, the difference between the conventional and rapid methods for ascorbic acid determination can be seen.

**Figure 8:**
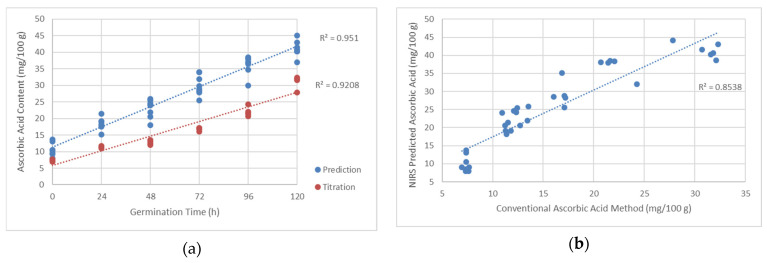
Ascorbic acid determination comparison between predicted titration and NIR spectroscopy: (**a**) Ascorbic acid content plot comparison for NIR spectroscopy predicted and titration values, and (**b**) ascorbic acid content plot of NIR spectroscopy predicted against titration values.

The comparison was also done in an objective-subjective plot (conventional titration method plotted against NIR spectroscopy predicted method) with R^2^ of 0.8538. Graphical representation can be seen in Figure 8 for a better perspective of the determination methods.

## 4. Discussion

### 4.1. Results of Conventional Methods

#### 4.1.1. Water Content

In mung beans, especially in sprouted ones, water content is one of the most important parameters, as water is essential for its growth. The amount of water that is used in hydrolytic reactions will represent the phase of growth and can be an indicator of the components that have been metabolized. Previous researchers indicated various ranges of water content in mung bean sprout within 0–5 days of germination time [14,24,45]. The water content changes within the first 72 h were described as a linear trend [46], and the result of the experiment agreed with the former research. The water content changes slowed down after 96 h of germination time, leading to a steeper growth within the curve. From the first 96 h, the development was also distinct in the two different growth phases, especially 12–36 h (S1) and 36–96 h (S2). This was due to the activation of hydrolytic enzymes, causing the bean to actively circulate the water from its environment for breaking down its inner components. A similar experiment was done to see the activity of α-amylase enzyme and the first 72 h period shows the highest activity of the mentioned enzyme [47]. There were two main phases established for the analysis and data evaluation during the 120 h period. The first phase corresponded to germination G1, it was presented during the first six hours and extended until twelve hours for some seeds. The seeds increased the water content from 47.94% to 55.6% (mean value), as can be seen in Figure 1. Seeds suffered a process of imbibition where they rapidly absorb water due to differences of osmotic pressure. Next, there was an activation of the metabolism, breathing process, and nutrients mobilization: in this stage, starch and proteins are converted into simple sugars and amino acids. The germination process is concluded after the embryo is elongated, the testa is ruptured, and the radicle has appeared [48,49].

The second phase, comprised of S1, S2, and S3, corresponded to the sprouting. It is characterized by the elongation of the radicle, described for a pale yellow to white color, and splitting of the cotyledons. The main factors for the elongation are the prior increase of extensibility of cell walls and activation of processes of elongation and relaxation of surrounding tissues with the hydrolysis of polysaccharides of the cell wall [48]. Some minor water content fluctuations were detected during the sprouting phase. Between S1 and S2 (36 and 48 h) and also S2 and S3 (90 and 96 h), there was a very small increase of water content; for the rest of the sprouting time, the water content increase steadily until 120 h, where the water content was around 85%.

#### 4.1.2. pH and Conductivity

One of the most affected compounds after the sprouting process in mung bean is phytic acid. Phytic acid is an anti-nutrient component that serves as the main phosphorous source of most legumes [7]. It is composed of an inositol ring and polyphosphate formation consisting of six phosphorous molecules. This molecule has high affinity with divalent ions such as zinc, calcium, and iron [50]. In this case, this molecule reduces the bioavailability of the mineral from bean consumption. Several processing and treatment methods have been reported to be effective to reduce the amount of phytate compounds within beans, such as cooking, soaking, and sprouting. Within the first 12 h of sprouting, there was a 12% reduction of this component [50], capping at 76% reduction from 72 h of sprouting [51]. While the reduction of this component will release free hydrogen ions, it also reduces its binding effect with the present mineral components. This results in lower pH measurement, and supposedly higher conductivity reading. Although the pH is quite straightforward, as it is only affected by the hydrogen ions, the conductivity is not that simple. Factors such as particle size and the conformation of the compounds can affect the conductivity reading [44]. The experiment results agree with the findings that the pH reading was a somewhat monotone trend of decreasing pH. Besides phytate components, the germination process also metabolizes more acidic components in the form of folate acid, ascorbic acid, and slightly acidic polyphenols [25,26]. Mung bean is a living organism that has high variability by itself, more so if another parameter (sprouting process) is taken into account.

When imbibition starts, reactivation of metabolism takes place when abscisic acid (ABA), responsible for dormancy, decreases, and gibberellins, gibberellic acid (GA), and ethylene increase. The increment of these vegetable hormones induces the activity of a great majority of amylolytic and proteolytic enzymes which have acid pH and are located in the endosperm. Hydrolysis of starch and proteins (converted in amino acids) is activated. Additionally, pH of the endosperm is acidic due to high malic acid concentration. The acid pH from malic acid lowers with germination. However, the acidity remains due to liberation of phosphoric acid and citric acid from aleuronal capes for gibberellic acid action. Moreover, lipids are degraded for lipases and glyoxysomes, producing fat acids and glycerol that contribute to the lowering of the pH [49].

According to previous research [52], which tested different times of imbibition for eggplant seed for the evaluation of physical quality, the conductivity increased with time as organic ions such as K^+^, Ca^2+^, Mg^2+^, Na^+^, and Mn^2+^, as well as sugars, amino acids, organic acids, and enzymes, are liberated as lixiviate. This fact can be indirectly related for measuring the conductivity when several compounds are solubilized and hydrolyzed during metabolism in germination. The conductivity increased from around 850 to around 1400 µS during the 120 h germination time. However, it denoted a positive correlation between conductivity and germination time. From the experiment, it can be said that while pH can give an approximation of the mung bean condition, conductivity measurement did not prove to be the best method for this assessment due to the high variability and conditions of the different beans.

#### 4.1.3. Ascorbic Acid

Ascorbic acid formation is one of the most significant features of the germination process. Aside from polyphenols, folic acid, and tocopherol [18,26,53], the formation of the other components are very low compared with ascorbic acid, thus it might be more difficult to develop a rapid technique for the other components. The redox titration method has been reported to show good accuracy in predicting ascorbic acid within the sub-percent level, even though the repeatability of the titration depends heavily on the selected titrant concentrations and proper standardization. The ascorbic acid determination based on titration showed a significant effect of germination within the 120 h interval of sprouting time, as can be seen in Figure 3.

The reason that the ascorbic acid metabolism peaks at the later part of germination is that the main material for ascorbic acid metabolism in the plant metabolism pathway relies heavily on the availability of D-glucose. After giving the bean enough time to sprout, the enzymes would have enough time to break down the polysaccharides into smaller monomer molecules, where it can be used for the ascorbic acid metabolism in its pathway. The breakdown of phytate components is also important, as there is a need for phosphorous molecules for the intermediate metabolites of ascorbic acid [54].

### 4.2. Near-Infrared Spectroscopy Measurement

For the NIR spectroscopy results of the mung bean sprout, the PCA plot of the whole spectra measurement (950–1630 nm) was observed to show less defined patterns than the ones with narrower spectra length (1300–1600 nm), as can be seen in Figure 2. The reason is that the latter wavelength was in the first overtone region of O-H bond, where most of the water molecules are detected. The water molecules are not strictly described as H_2_O, but also the water molecule species [36]. Observing this region can give us information of covalent O-H and hydrogen bonds that are highly sensitive and would reflect even the smallest hint of molecular perturbation [34]. Although the conventional NIR spectroscopy method that involves the whole range of near-infrared wavelengths has been commonly used for quality purposes in mung bean and mung bean sprouts [27,55], comparison with the aquaphotomics-specific method can be interesting to view, and the important wavelengths can be seen in Table 2.

From the scans, it could be seen that some important regions within the NIR wavelength contributed to the different principal component scores. The absorption bands located around 1024 nm could be associated with O-H stretch second overtone of starch (or sugars). The absorption around 1047 nm is typical for the second overtone of N-H bond (CONH) [56]. The region of second overtone for C-H (1100–1225 nm) and combination of C-H (1300–1420 nm) were considered important in both bean sprout and bean sprout extract samples [57]. The changes within a larger polysaccharides molecule would be detected in these wavelength regions. Besides that, the importance of the first region of O-H overtone (1300–1600 nm) can be seen, where it contributed to a significant number of the important wavelengths. An interesting point of view is that the important wavelength shifts a bit for the bean sprout sample after truncating the wavelength of interest, but the ones that were prepared in liquid form showed better stability, showing almost the same absorbance importance when analyzed with either truncated or full spectra. Aquaphotomics that specifically examines the first overtone region defines 12 so-called water matrix coordinates (WAMACs) under the first overtone of OH, which are the spectral ranges where water-specific absorption bands are most likely to be found [58]. The absorption band at 1381 nm coincides with the C4 coordinate corresponding to the OH –(H_2_O)n (*n* = 1, 4) water solvation shell, while 1391, 1393, 1396, and 1403 nm wavelengths indicate trapped water within the C5 coordinate [59] or free water molecules [60]. The absorption at 1454 nm falls into the range of the C8 coordinate, which signifies the OH –(H_2_O)n (*n* = 4, 5) water solvation shell. This band was found typical in all cases. The larger wavelengths are in the range of C9–C12 coordinates, which denote water molecules increasingly bound by hydrogen bonding [61]. However, wavelengths above 1500 nm could be associated with N-H stretch first overtones [57]. As mung bean is an organism that is expected to be changing, generating a general sample in the form of aliquots is preferred. When such aliquots are present, then continuing the analysis using an aquaphotomics approach is beneficial in many ways. The models discussed will be based on these aliquots.

For the classification, it can be seen in Figure 2 that there was no significant trend for the intact mung bean scans. While NIR spectroscopy is commonly used for in-line quality checks in such an industry, it can easily differentiate between significant features that are off from the product, typical of broken seeds [62]. Although, in the form of aliquots (Figure 3), the classification had better between-group variance, and is easily differentiable. The analysis was then continued with a discriminant analysis (Figure 4), where it shows even better within-group variance that was lacking in the previous score plots using PCs. The LDA model can classify the bean sprouts based on the germination time of 24 h intervals at 100% accuracy.

For the PLSR of the bean sprout extract, it showed great accuracy in predicting the sprouting time and water content. The important wavelengths of water content prediction align well with the ones for sprouting time determination. It confirms that as water is a major component, it can be used to differentiate the bean based on the germination time. As summarized in Table 2 the wavelengths corresponding to the C2, C5, C6, C8, C10, and C12 water matrix coordinates contributed the most to the predictions of ascorbic acid. These wavelength ranges are assigned to OH– (H_2_O)n (*n* = 1, 2, 4) hydroxylated water clusters, to trapped water, water molecules involved in hydration, to OH –(H2O)n (*n* = 4, 5) water solvation shell, and to strongly bound water molecules [58,59]. Wavelengths 1519 and 1536 nm can be associated with first overtone of N-H bands, which can confirm the changes in proteins during germination [63]. Besides the influence of N-H bands, the wavelength around 1500 nm is also associated with strongly bound water molecules [60], which showed changes during the germination process (Figure 7a).

### 4.3. Ascorbic Acid Modelling and Prediction

The PCA plot of pure ascorbic acid showed some within-class variance in the respective groups of concentrations. Although there has been many researches that can easily detect ascorbic acid in this range of concentration [55,64,65], the case might be more difficult in the form of more complex tissue. Despite the low concentration, the PCA plot shows some monotone trends that classify each concentration in a distinguishable manner. The PLSR plot showed great capability of ascorbic acid detection within the 0–50 mg/100 g concentration range, and important wavelengths were noted at 1414, 1477, and 1503 nm for ascorbic acid (Figure 6c). The important wavelengths can be easily matched with the wavelength from the bean sprout extract, and it can be recognized that the spectral data at 1393–1414, 1454–1477, and 1503–1522 nm match with the bean sprout extract important wavelengths. These wavelengths can also be observed in the plotted aquagram (Figure 7a), where the peaks can be observed at 1462, 1477, 1489, and 1513 nm for the strong bonded water [61]. From the aquagram, it can be agreed that there was a tendency of forming stronger bonded water molecules. For sprouting, activation of hydrolytic enzymes as well as the formation of smaller molecules would release water as its by-product, allowing more water molecules to be released from the system. Some of the components are strongly hydrophilic, thus increasing the presence of strongly bonded water molecules [66]. For ascorbic acid, it is considered as a reducing agent, thus normally it reduces oxygen into water and has a great solubility in water [67,68]. There was also a suggestion that in living matrixes, the arrangement of the components would have a tendency to create efficient inter-molecular hydrogen bonds if there were plenty of hydrogen acceptors/donors in the system. This phenomena was observed and considered common in biological tissues, and named as ‘collectivity’ [59].

Prediction of ascorbic acid content in bean sprout, which is considered a complex tissue, was seen to be possible. A similar experiment was conducted in another complex matrix of baby food, and it detected ascorbic acid within the sub-percent successfully from the mixtures [67], with adequate R^2^ of 0.81. In case of the aquaphotomics approach, the coefficient of determination is higher from the result (Figure 7), and the results were matched with ascorbic acid determination using conventional means. The trend of the ascorbic acid agreed with the results from titration (Figure 8), although a shifting tendency from the rapid analysis and the conventional method can be seen. The rapid analysis recorded values that are constantly higher compared to the ones from the titration method. Within the sprouted beans, there was also the metabolism of many active components such as tocopherol and polyphenols [8,53]. The mentioned components might show similar features in absorbance within the important wavelengths for the ascorbic acid prediction. The difference between predicted ascorbic acid content and conventional determination can also be considered from the biochemical point of view. As the bean underwent germination, larger polysaccharides were broken down into smaller molecules of saccharides [26]. In this case, the amount of hydroxyl functional group in the matrix increased exponentially according to the germination dynamics. Future study will be needed to identify the metabolites of germination and the possibility of detection using NIR spectroscopy. Although, if the shift is persistent and systematic, the model can easily be adjusted to comply with the analytical results. For now, it seems that NIR spectroscopy is potent enough to offer more information rather than simpler analysis or high/low ascorbic acid content sorting. Considering the results from NIR spectroscopy analysis, a similar approach could be taken with other plant crops for quality evaluation purposes. Although the component of interest might not necessarily be ascorbic acid, other relevant metabolites could be chosen as an indicator of other plant growth observations.

## 5. Conclusions

The results of the ANOVA test showed a significant effect of germination time in the water content of the sprouted beans (*p* < 0.05). The post-hoc test revealed that each of the germination time points were significantly different from each other. Germination time showed a significant effect on both the pH and conductivity of the bean sprout extract sample (*p* < 0.05). There was poor separation of the germination time points in the PCA for both the bean sprout and bean extract when the wavelength range 950–1650 nm was used. Contrarily, there was a good separation of all the germination time points when the first O-H overtone range was used, suggesting that this wavelength range may be the best option for distinguishing mung bean germination times. For this separation, the wavelengths 1024, 1046, 1147, 1196, 1226, 1381, 1403, 1454, and 1568 nm were found to be the most relevant for discriminating the bean sprout when the wavelength range 950–1650 nm was used, and it was 1391, 1403, 1460, and 1562 nm when the first O-H overtone range, i.e., 1300–1600 nm, was used. For the bean sprout extract, it was 1092, 1157, 1225, 1265, 1393, 1396, 1454, 1522, and 1545 nm when the wavelength range 950–1650 nm was used, and it was 1393, 1396, 1454, 1522, and 1545 nm when the first O-H overtone was used. All the different germination time points could be classified with 100% correct accuracy for the bean sprout and extracts using the wavelength range 950–1650 nm and also the first O-H overtone. PLSR regression models showed high accuracies even after cross-validation (R^2^CV) and low errors (RMSECV) for predicting water content, germination time, and ascorbic acid. Aquagrams from aquaphotomics evaluations showed similarities in trend. Both mung bean sprout and ascorbic acid initially showed the abundance of weaker bonded water molecules (first and fourth quadrant), and stronger bonded water (1513 nm) was observed under higher concentrations of ascorbic acid and longer germination time. From our findings, water content is indeed a prominent indicator for bean sprouting and contributes to the prediction of germination time of the bean. pH and conductivity parameters were deemed not favorable for mung bean sprout observation, as there is fluctuating changes within the system. NIR spectroscopy has shown potential for complex biochemical monitoring of mung bean germination and detection of ascorbic acid content within the sub-percent level. Conventional ascorbic acid determination requires extract preparation of the samples and titration reagents. NIR-based ascorbic acid prediction with extract preparation for aquaphotomics analysis is a viable option that could be adopted for monitoring mung bean quality in the legume industry, as it is more beneficial in efficiency and from an economical point of view.

## Figures and Tables

**Table 1:** Water content, pH, and conductivity values of mung bean sprouts.

Germination Time (h)	Water Content (%)	pH	Conductivity (µS/cm)	Growth Stage
0	47.94 ± 0.38 ^a^	6.41 ± 0.01 ^a^	837 ± 5.57 ^a^	G1
6	55.61 ± 0.36 ^b^	6.43 ± 0.02 ^a^	851 ± 2.65 ^b^	G1
12	56.54 ± 0.39 ^c^	6.41 ± 0.02 ^ab^	899 ± 4.00 ^c^	S1
18	59.62 ± 0.41 ^d^	6.30 ± 0.02 ^b^	910 ± 4.73 ^d^	S1
24	61.61 ± 0.31 ^e^	6.37 ± 0.03 ^bc^	947 ± 4.58 ^e^	S1
30	63.86 ± 0.34 ^f^	6.25 ± 0.03 ^d^	992 ± 6.08 ^g^	S1
36	64.50 ± 0.31 ^g^	6.13 ± 0.03 ^e^	831 ± 6.67 ^a^	S1
42	64.76 ± 0.28 ^h^	6.17 ± 0.03 ^e^	1005 ± 7.00 ^h^	S2
48	67.44 ± 0.39 ^i^	6.33 ± 0.01 ^b^	1030 ± 6.08 ^i^	S2
54	69.01 ± 0.47 ^j^	6.07 ± 0.02 ^f^	981 ± 4.16 ^f^	S2
60	70.86 ± 0.36 ^k^	6.03 ± 0.03 ^g^	1201 ± 6.67 ^l^	S2
66	73.69 ± 0.36 ^l^	5.88 ± 0.03 ^h^	951 ± 4.16 ^e^	S2
72	75.98 ± 0.39 ^m^	5.55 ± 0.03 ^i^	1139 ± 4.58 ^j^	S2
78	78.14 ± 0.46 ^n^	5.55 ± 0.01 ^i^	1433 ± 4.36 ^p^	S2
84	79.48 ± 0.31 ^o^	5.84 ± 0.03 ^h^	1251 ± 5.20 ^m^	S2
90	80.67 ± 0.37 ^p^	5.22 ± 0.01 ^k^	1131 ± 6.51 ^j^	S2
96	81.15 ± 0.33 ^q^	5.34 ± 0.03 ^j^	1514 ± 6.43 ^r^	S3
102	82.33 ± 0.44 ^r^	5.19 ± 0.03 ^k^	1157 ± 3.06 ^k^	S3
108	83.80 ± 0.46 ^s^	5.19 ± 0.03 ^k^	1302 ± 6.51 ^n^	S3
114	83.95 ± 0.33 ^t^	5.07 ± 0.03 ^l^	1492 ± 6.03 ^q^	S3
120	85.24 ± 0.44 ^u^	5.06 ± 0.03 ^l^	1354 ± 1.73 ^o^	S3

Values are represented in Mean ± standard deviation. Different superscript letters (a–u) denote significant difference based on results from analysis of variance (ANOVA) followed by Duncan’s Multiple Range Test (DMRT) for each germination time.

**Table 2:** Important wavelengths from the NIR spectroscopy measurement for bean sprout and bean sprout extract.

Sample	Wavelength	Important Wavelengths (nm)
Bean sprout	950–1630 nm	1024, 1046, 1147, 1196, 1226, 1381, 1403, 1454, 1568
	1300–1600 nm	1391, 1403, 1460, 1562
Bean sprout extract	950–1630 nm	1092, 1157, 1225, 1265, 1393, 1396, 1454, 1522, 1545
	1300–1600 nm	1393, 1396, 1454, 1522, 1545

**Table 3:** Ascorbic acid value of bean sprout based on conventional and rapid methods.

Germination Time (h)	Titration (mg/100 g)	Prediction (mg/100 g)
0	7.31 ± 0.34 ^a^	10.48 ± 2.49 ^a^
24	11.32 ± 0.43 ^b^	18.51± 2.37 ^b^
48	12.86 ± 0.61 ^b^	23.40 ± 2.19 ^b^
72	16.74 ± 0.60 ^c^	29.85± 3.17 ^c^
96	21.44 ± 0.69 ^d^	36.43 ± 2.87 ^d^
120	30.58 ± 2.39 ^e^	40.97 ± 1.84 ^d^

Values are represented in Mean ± standard deviation. Different superscript letters (a–e) denote significant difference based on results from ANOVA followed by DMRT for each germination time.

## Data Availability

The data presented in this study are available on request from the corresponding author. The data are not publicly available due to privacy and ethical reasons.
